# The Novel Somatosensory Nose-Poke Adapted Paradigm (SNAP) Is an Effective Tool to Assess Differences in Tactile Sensory Preferences in Autistic-Like Mice

**DOI:** 10.1523/ENEURO.0478-22.2023

**Published:** 2023-08-28

**Authors:** Matthew S. Binder, Angelique Bordey

**Affiliations:** Departments of Neurosurgery and Cellular and Molecular Physiology, Wu Tsai Institute, Yale School of Medicine, New Haven, CT 06520-8082

**Keywords:** autism, behavior, locomotion, novel methodology, somatosensory deficit, stereotypy

## Abstract

One of the most prevalent deficits in autism spectrum disorder (ASD) are sensitivities to sensory stimuli. Despite the prevalence of sensory deficits in autism, there are few paradigms capable of easily assessing sensory behaviors in ASD-like mouse models. We addressed this need by creating the Somatosensory Nose-poke Adapted Paradigm (SNAP), which consists of an elevated platform with 6 holes in the center, half of which are lined with sandpaper and half are smooth, requiring mice to use their whiskers to sense the texture. The SNAP paradigm assesses tactile sensory preferences as well as stereotypy, anxiety, and locomotion. We used two wild-type (neurotypical) mouse strains, C57BL/6J (C57) inbred and CD-1 outbred mice, and two ASD mouse models, BTBR (a model of idiopathic ASD) and *Cntnap2*^−/−^ mice (a model of syndromic ASD). We found that both ASD models produced more nose pokes into the rough condition than the smooth condition, suggesting an increased preference for complex tactile stimulation when compared with the neurotypical groups, wherein no differences were observed. Furthermore, we found increased stereotypy and time spent in the center, suggestive of decreased anxiety, only for BTBR mice compared with the other mouse strains. Overall, SNAP is an easy to implement task to assess the degree of preference for complex tactile stimulation in ASD mouse models that can be further modified to exclude possible confounding effects of novelty or anxiety on the sensory preferences.

## Significance Statement

Despite sensory deficits occurring in 90% of individuals with autism spectrum disorder (ASD), there are few behavioral sensory tasks available. To address this need, we developed a tactile sensory task, called the Somatosensory Nose-poke Adapted Paradigm (SNAP) that harnesses innate behavior, is easy to implement, and is not memory dependent. We assessed two neurotypical mouse strains: C57 and CD-1 mice, and two ASD mouse models: BTBR and *Cntnap2*^−/−^ mice. Both ASD models displayed preferences for rough textures and interstrain differences in stereotypy, anxiety, and locomotion. SNAP is thus an easy to implement test to assess differences in tactile sensory preferences in ASD mouse models.

## Introduction

Autism Spectrum Disorder (ASD) is a heterogenous neurodevelopmental disorder that is found in one out of 44 children (Maenner et al., 2021). The clinical diagnosis of ASD is based on patterns of behavior that include socio-communicative deficits, repetitive movements, and may also include abnormalities in response to sensory stimulation (American Psychiatric Association, 2013). Most of the research in both mice and humans has focused on the social and communicative deficits of ASD. Therefore, sensory abnormalities are vastly understudied in ASD despite their occurrence in 90% of patients (Robertson and Baron-Cohen, 2017; Mikkelsen et al., 2018; Balasco et al., 2019; Dellapiazza et al., 2020). Furthermore, sensory abnormalities have been shown to exacerbate existing social abnormalities and worsen the autistic phenotype (Marco et al., 2011; Balasco et al., 2019; Dellapiazza et al., 2020). Thus, there is a significant need for a greater understanding of sensory behaviors in ASD as they may provide a gateway to a better overall understanding of autism.

A leading reason why sensory behaviors are understudied in murine models is the lack of an easy to implement and effective sensory behavioral task, as most of the available tactile tasks are complex and have significant drawbacks. For instance, in one task mice are restrained and discriminate between different textures using their whiskers, whereas in another, mice are placed in a maze that they navigate based off of tactile cues (Lipp and Van der Loos, 1991; Guic-Robles et al., 1992; O’Connor et al., 2010). Both tasks require numerous training trials per animal, take significant time, and can stress the animal. Considering these shortcomings, two research teams modified the novel object recognition (NOR) task to assess sensory behaviors. Wu et al. (2013) focused on tactile discrimination between various grits of sandpapers, whereas Orefice et al. (2016) examined sensory preference in ASD models using rough and smooth blocks. Although these modifications simplified the tasks, several disadvantages remain. For instance, both tasks are based off a memory paradigm, therefore a learning or memory impairment may complicate assessment. This is particularly relevant in ASD, as numerous ASD models have learning and memory deficits (Peñagarikano et al., 2011; Amodeo et al., 2012; McTighe et al., 2013; Orefice et al., 2016). Additionally, the novel object recognition-based tasks are labor intensive, requiring several days of training. Lastly, the tasks either assessed only glabrous skin, as the whiskers were surgically removed before the trial (Orefice et al., 2016), or lack tactile precision, as the mice could interact with the objects using both whiskers and paws (Wu et al., 2013). Since whiskers are the principal tactile sensory organ in mice and most analogous to fingertips, the human primary tactile sensory organ, studies preferentially assessing whisker sensitivities may have increased generalizability (Diamond et al., 2008; Adibi, 2019; Warren et al., 2021).

In light of these limitations, we developed a novel tactile based paradigm, the Somatosensory Nose-poke Adapted Paradigm (SNAP), that does not require training, animal restraint, or surgery, is not memory-dependent, isolates whiskers for tactile assessment, and is easy to implement and cost/time effective. Since mice make and explore holes in the ground to seek shelter or food, we created a paradigm that harnesses this innate behavior and allows mice to explore premade holes (by poking their noses into them, hence the term nose-poke) that were lined with different textures. This forced the mice to brush their whiskers across either textured or smooth holes each time they made a nose poke. The number of nose pokes made in smooth and textured holes was recorded along with the total number of nose pokes made overall, the total distance traveled, and the time spent in the center region of the chamber. We used the SNAP paradigm to assess potential differences in tactile sensory preferences in a classic idiopathic model of ASD (BTBR mice) as well as in a prominent syndromic model of ASD (*Cntnap2*^−/−^ mice) and compared them to C57 inbred and CD-1 outbred neurotypical mice. Our novel task detected increased preference for complex tactile stimulation in ASD-like mice when compared with neurotypical mice. This newly developed task together with other behavioral assays may help to better phenotype ASD mouse models.

## Materials and Methods

### Subjects

C57BL/6J (C57), B6.129(Cg)-*Cntnap2tm1Pele*/J (*Cntnap2*^−/−^ mice), and BTBR T+ Itpr3tf/J (BTBR) mice were purchased from The Jackson Laboratory, whereas CD-1 mice were purchased from Charles River. These strains were chosen to assess the validity of our behavioral task in inbred (C57) and outbred (CD-1; neurotypical) mice as well as in an idiopathic (BTBR) and a syndromic (*Cntnap2*^−/−^) ASD model. A total of 81 mice were used: 21 C57BL/6J (11 males, 10 females), 18 CD-1 (nine males, nine females), 22 *Cntnap2*^−/−^ (11 males, 11 females), and 20 BTBR mice (10 males, 10 females). Animals were tested during the light cycle, between 1 and 4 P.M. and were five to six weeks old. The mice were group-housed in a climate-controlled colony room on a 12/12 h light/dark cycle with *ad libitum* access to food and water. All test procedures were conducted in compliance with the National Institutes of Health *Guidelines for the Care and Use of Laboratory Animals* and were approved by Yale University’s Institutional Animal Care and Use Committee.

### Experimental design for SNAP

**Figure 1.**
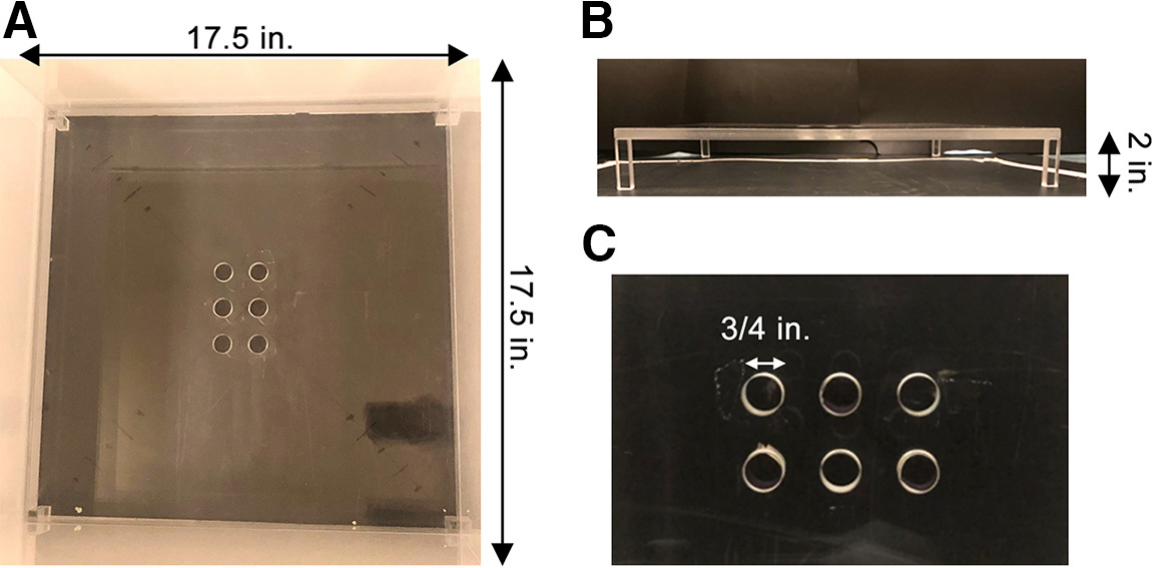
Somatosensory Nose-poke Adapted paradigm (SNAP). ***A***, Photograph illustrating the SNAP chamber consisting of a 17.5 inch (W) × 17.5 inch (L) × 24 inch (H) acrylic, open box. ***B***, ***C***, Photograph of the 2-inch elevated platform contained in the SNAP chamber. The platform has six ¾ inches in diameter holes in the center. Half of the holes are lined with 80 grit sandpaper whereas the other half do not.

Mice were habituated to the testing room for 30 min. They were then individually removed from their home cage and placed on a clear elevated platform (2 inches high) that was made out of acrylic. The platform had six ¾ inches in diameter holes in the center with a depth of 1 cm (Fig. 1*A–C*). Half of the holes were lined with 80 grit coarse sandpaper (3M Pro Grade Precision), constituting a rough condition, whereas the other half were not lined, constituting a smooth condition. The sandpaper was precisely cut so it exactly fit each hole with no overlap. The pressure of the sandpaper on the hole was sufficient to secure it so no glue was necessary. The holes lined with sandpaper were randomized between trials. Tape was attached underneath the elevated platform to differentiate smooth from rough holes to the experimenter. The sandpaper was replaced with new sandpaper between testing days. The platform was contained within a 17.5 inch (w) × 17.5 inch (l) × 24 inch (h) testing chamber made of opaque acrylic (Fig. 1*A*). The platform formed the bottom of the chamber, so mice were unable to crawl under the elevated platform. The mice were allowed to explore the chamber for 5 min and were video-recorded with a high-definition IP camera (MegaVideo AV2115DNAIv1, Arecont Vision) at a 20-Hz acquisition rate that was mounted directly above the test chamber. Following testing, the mice were removed from the chamber and placed into a clean holding cage until all of the mice had been tested, at which point they were returned to their home cage. The apparatus was cleaned with 70% isopropyl alcohol between trials to eliminate any sensory cues. A trained experimenter blind to the condition of the animal scored the video, recording the number of nose pokes into the rough and smooth holes. A hole poke consisted of the mouse poking their entire nose into the hole (up to their eyes), any partial pokes were not scored. The total distance ran by each mouse and the time spent in the center (8 × 8 inches) region were calculated using the ANY-maze software.

### Statistical analysis

All data were analyzed using IBM SPSS Statistics 21.0 (IBM) or GraphPad Prism 7 software. A repeated measures ANOVA was used to analyze differences in sensory preferences which was followed by Sidak *post hoc* tests to clarify any significant interactions. ANOVA tests were used to assess stereotypy (the total number of nose pokes produced), distance traveled, and the duration spent in the center of the apparatus (an 8 × 8 inch region). Tukey’s HSD *post hoc* test was used to clarify any significant interactions for the ANOVAs. A value of *p *<* *0.05 was considered significant for each statistical test, with figures depicting the mean ± SEM.

## Results

### Behavioral sensory preference

**Figure 2.**
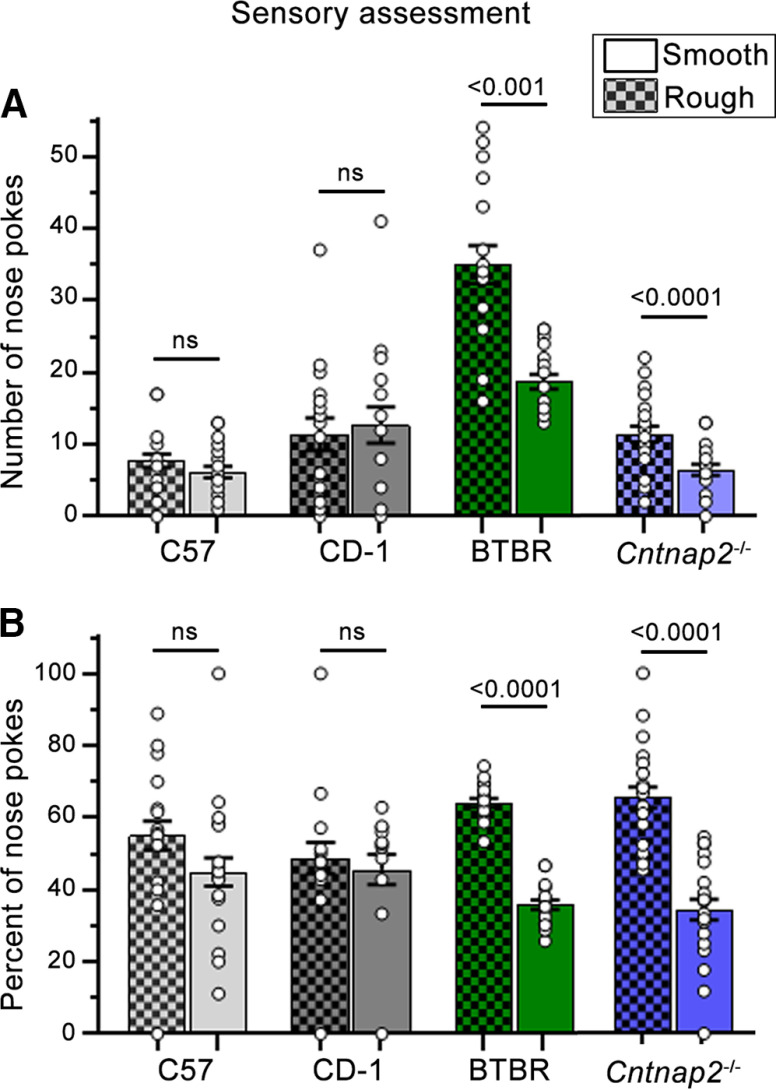
Behavioral sensory assessment. ***A***, Bar graphs of the number of nose pokes into rough and smooth holes, BTBR and *Cntnap2*^−/−^ produced more rough than smooth nose pokes relative to neurotypical mice, *p* < 0.001. ***B***, Bar graphs of the percentage of nose pokes into rough and smooth holes, BTBR and *Cntnap2*^−/−^ produced a higher percentage of rough than smooth nose pokes relative to neurotypical mice, *p* < 0.0001. Data are represented as the mean ± SEM.

C57BL/6J and CD-1 neurotypical mice and *Cntnap2*^−/−^ and BTBR ASD-like mice of similar ages were assessed with the SNAP paradigm. They were tested in a chamber containing an elevated platform with six ¾ inches in diameter holes in its center. Half of the holes were lined with 80 coarse-grit sandpaper to create a rough texture and half of the holes remained smooth. Abnormalities in response to sensory stimulation were assessed by quantifying the number of nose pokes made in rough and smooth holes. When assessing sensory preference, we found a main effect for texture (*F*_(1,73)_ = 78.59, *p* < 0.001), a main effect of strain (*F*_(3,73)_ = 34.98, *p* < 0.001) and a texture by strain interaction *F*_(3,73)_ = 39.01, *p* < .001. No main effect of sex was found (*F*_(1,73)_ = 0.36, p = . 55) nor was there a texture by sex (*F*_(1,73)_ = 0.06, *p* = 0.81), strain by sex (*F*_(3,73)_ = 0.09, *p* = 0.97) or texture by strain by sex (*F*_(3,73)_ = 0.36, *p* = 0.78) interactions. Male and female data were thus pooled. Sidak *post hoc* tests found that BTBR and *Cntnap2*^−/−^ mice produced significantly more nose-pokes into rough holes than smooth holes (BTBR *p* < 0.001, *Cntnap2*^−/−^ *p* < 0.0001), whereas both C57 and CD-1 mice displayed no preference (*p* > 0.05; Fig. 2*A*).

Considering the differences in the number of nose-pokes between mouse strains and to better illustrate the interstrain texture preferences, we calculated the percentage of nose pokes made in each condition per mouse. We ran a repeated measures ANOVA and found a main effect of texture (*F*_(1,73)_ = 36.04, *p* < 0.001) and a texture by strain interaction (*F*_(3,73)_ = 4.96, *p* = 0.003). No main effect of strain (*F*_(3,73)_ = 1.18, *p* = 0.32) nor sex (*F*_(1,73)_ = 1.27, *p* = 0.26) were found. There were also no texture by sex (*F*_(1,73)_ = 0.35, p = . 56), strain by sex (*F*_(3,73)_ = 1.18, *p* = 0.32), texture by strain by sex (*F*_(3,73)_ = 1.26, *p* = 0.30) interactions. Sidak *post hoc* analyses found that BTBR and *Cntnap2*^−/−^ mice both produced a higher percentage of rough nose pokes than smooth nose pokes (*p* < 0.0001) while no differences were found between C57 and CD-1 mice (*p* > 0.05; Fig. 2*B*). These data suggest that BTBR and *Cntnap2*^−/−^ mice display a tactile sensory alteration compared with neurotypical mice.

### Stereotypy assessment

**Figure 3.**
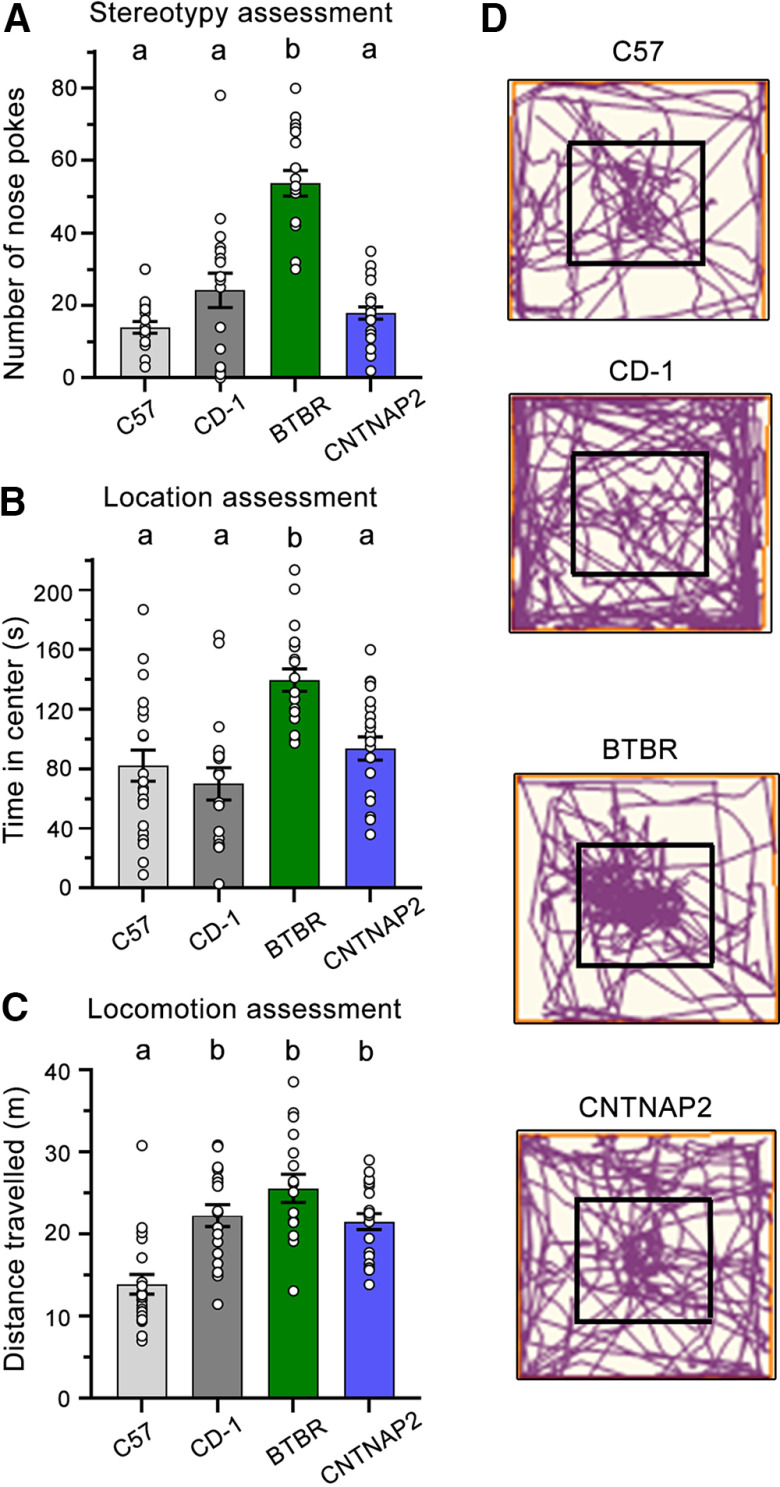
Stereotypy, anxiety, and locomotion assessment in SNAP. ***A***, Bar graphs of the total number of nose pokes for each mouse line. BTBR mice generated more nose pokes than all other lines, indicative of increased stereotypy (difference between A and B notations for stereotypy, *p* < 0.0001). ***B***, Bar graphs of the time spent in the center of the paradigm, a measure of anxiety. BTBR mice spent more time in the center than all other groups (difference between A and B notations for anxiety, *p* < 001). ***C***, Bar graphs of the distance traveled, a measure of locomotion. C57 mice traveled significantly less than all other groups (difference between A and B notations for locomotion *p* < 0.0002). ***D***, Representative track plots of C57, CD-1, *Cntnap2*^−/−^, and BTBR mice. Data are represented as the mean ± SEM.

The SNAP paradigm can also be used to assess stereotypy or the repetitive behavior of the mice, another key aspect of ASD. To do this, the total number of nose pokes per group was summed. We found a main effect of strain (strain: *F*_(3,73)_ = 34.98, *p* < 0.001) but no main effect of sex (*F*_(1,73)_ = 0.36, *p* = 0.55), nor any strain by sex interaction (*F*_(3,73)_ = 0.09, *p* = 0.97). Tukey’s HSD *post hoc* analyses revealed that BTBR mice produced more total nose pokes than any other group (*p* < 0.0001). No differences were found between *Cntnap2*^−/−^, CD-1, and C57 mice (*p* > 0.05; Fig. 3*A*).

### Duration in the center of chamber

We next analyzed the duration of time spent in the center of the arena, an 8 × 8 inch region around the holes, to ensure that all the mice interacted with the holes and to provide an approximation of anxiety. We found a main effect of strain (strain: *F*_(3,73)_ = 10.56, *p* < 0.001) but no main effect of sex (*F*_(1,73)_ = 0.02, *p* = 0.90) nor any strain by sex interaction (*F*_(3,73)_ = 1.20, *p* = 0.32). A Tukey’s *post hoc* test found that BTBR mice spent significantly more time in the center of the chamber than the other groups (BTBR vs *Cntnap2*^−/−^, *p* < 0.001, BTBR vs CD-1, *p* < 0.0001, BTBR vs C57, *p* < 0.001). There were no differences in time spent in the center for C57, CD-1, and *Cntnap2*^−/−^ mice (*p* > 0.05; Fig. 3*B*).

### Distance traveled

Lastly, the total distance traveled in both groups was assessed to verify that all the mice explored the chamber and to provide an approximation of each group’s activity levels. We found a main effect of strain (*F*_(3,73)_ = 13.62, *p* < 0.001) but no main effect of sex (*F*_(1,73)_ = 0.64, *p* = 0.43) nor any strain by sex interaction (*F*_(3,73)_ = 0.011, *p* = 0.99). Tukey’s HSD *post hoc* analyses found that C57 mice traveled a significantly shorter total distance than all other groups (C57 vs CD-1, *p* < 0.001, C57 vs *Cntnap2*^−/−^, *p* < 0.001, C57 vs BTBR, *p* < 0.0001). No other differences between strains were found (*p* > 0.05; Fig. 3*C*). Representative track plots of each model are depicted in Figure 3*D*.

## Discussion

Here, we describe a novel sensory paradigm, the Somatosensory Nose-poke Adapted Paradigm (SNAP). SNAP utilizes natural mouse behaviors to efficiently assess differences in tactile sensory preferences, with an emphasis on complex sensory stimulation (i.e., sandpaper). Using SNAP, we investigated the behavior of ASD-like mice (BTBR and *Cntnap2*^−/−^) that are widely used ASD behavioral models with strong, but not identical, ASD-like phenotypes. Furthermore, BTBR mice are a model of idiopathic autism whereas *Cntnap2*^−/−^ mice are a model of syndromic autism (McFarlane et al., 2008; Scattoni et al., 2008; Silverman et al., 2010).

Using SNAP, we found that both ASD models displayed a nearly 2-fold preference for a rough texture versus a smooth texture, whereas both C57 and CD-1 strains showed no preference. These data suggest that both BTBR and *Cntnap2*^−/−^ mice have an increased preference for complex tactile sensory stimulation compared with neurotypical mice. One interpretation of these findings is that the two ASD mouse models have abnormalities in tactile sensory information processing compared with neurotypical mice. An alternate interpretation is that the rough condition may represent novelty and thus animals would prefer the novel rough condition that they have not been exposed to. Although BTBR mice have been shown to display novelty aversion (McTighe et al., 2013), their response to novelty is unknown in the paradigm used here. It is also unknown for *Cntnap2*^−/−^ mice. It may thus be important to limit the confounding effect of novelty. To do this, the experimenter could increase the amount of time for exploration (1 h instead of 5 min) and examine whether the sensory preference remains or goes away; another option is to have a 1-h-long habituation phase (either the same day or an earlier day) with the holes and the different textures before testing. The novelty effect and the design of the apparatus (holes in the center of the platform) could also trigger anxiety and affect the way animals perceive and respond to their environment as well as the amount of time spent in the middle of the arena. We found that BTBR mice spent a large amount of time in the center of the chamber, suggestive of decreased anxiety. This finding is consistent with previously reported decreased anxiety in BTBR mice using a different task (Pobbe et al., 2011). By contrast, *Cntnap2*^−/−^ mice were indistinguishable from C57 mice in measures of anxiety as previously reported using a different task (Brunner et al., 2015; Sacai et al., 2020). One option to mitigate the effect of anxiety would be to have the holes uniformly distributed in the chamber and placed the platform in a dark enclosure with a hole in the ceiling for an infrared camera.

We also reported increased stereotypy for BTBR mice but not for *Cntnap2*^−/−^ mice based on the number of total nose pokes, consistent with previous reports of stereotypy for these mouse strains (Amodeo et al., 2012; Brunner et al., 2015; Xing et al., 2019). For the BTBR mice, it is possible that the increased stereotypy was partially because of an increased opportunity to make nose-pokes since these mice remained longer in the center of the platform. Uniformly distributing the holes throughout the chamber as suggested above would address this issue. Finally, we assessed locomotion and found that while BTBR and *Cntnap2*^−/−^ mice traveled significantly more than C57 mice, the total distance traveled in ASD models was not different from CD-1 mice. Despite some of the shortcomings listed above, our novel task, SNAP, offers a reliable assessment of tactile sensory preferences, repetitive, anxiogenic, and locomotor behaviors in an idiopathic and a syndromic ASD mouse model. The proposed modifications would help exclude possible confounding effects of novelty and anxiety on the sensory preferences. This would allow us to conclude that the observed differences in tactile sensory preferences were primarily because of alterations in sensory processing.

In humans, ASD-associated sensory deficits can be grouped into two overarching categories: hypersensitive, defined as an exaggerated response to a sensory stimulus that leads to stimulus avoidance, or hyposensitive, defined as an interest in experiences that are prolonged or intense which leads to sensation seeking behavior (Dunn, 1997; Ben-Sasson et al., 2009). Studies have found that individuals with tactile hyposensitivity will excessively touch objects in their environment to increase sensation (Foss-Feig et al., 2012; Kellaher, 2015; Mikkelsen et al., 2018). This closely resembles what we observed in our study, as both ASD models produced more nose pokes in the rough condition than the smooth condition, suggestive of a sensation seeking behavior. However, our task cannot identify the exact somatosensory mechanism (e.g., hyposensitivity of tactile function or increased stimulation-induced repetitive behavior) responsible for the sensory preference, that would require additional stimulation paradigms.

Currently there are few murine behavioral sensory paradigms available, and the existing paradigms have significant limitations compared with SNAP. Both previously described behavioral sensory paradigms are variations on the novel object test (NOR), which is principally an assessment of memory. Several murine models of ASD, such as *Fmr1*, *Mecp2*, *Shank3*, *Cntnap2*^−/−^, and BTBR mice are known to have deficits in memory and in behavioral flexibility in learning tasks, which could impact the NOR-based assessment of sensory behavior (Peñagarikano et al., 2011; Amodeo et al., 2012; McTighe et al., 2013; Orefice et al., 2016). SNAP utilizes an instinctual foraging behavior of mice that is not memory dependent, avoiding this potential confound. Both NOR tasks also require significant time, as one paradigm consists of 2 habituation days and a test day (totaling 33 min per mouse, not including analysis; Wu et al., 2013), whereas the other consists of 2 habituation days and 3 test days with two session per day (totaling 95 min per mouse, not including analysis; Orefice et al., 2016). Conversely, SNAP is conducted in one 5-min-long session followed by a 5 min analysis, making it at least three to nine times faster than the other paradigms. Even after adding a habituation day or longer recording time, SNAP would be significantly shorter than the other tasks. Lastly, the NOR-based tasks are less precise than SNAP in terms of the sensory organs tested. Previous studies either examined tactile sensation via glabrous skin (after surgical removal of the whiskers) or via both the skin (paws) and whiskers (Wu et al., 2013; Orefice et al., 2016). In comparison, SNAP localizes the tactile sensory input to the vibrissae, as only the mouse’s head goes into the holes, thus only the whiskers brush against the different textures. This distinction is important because whiskers are considered to be the specialized touch organs of mice (not glabrous skin) and are the equivalent of fingertips in primates for tactile sensation suggestive of a strong interspecies applicability of SNAP (Diamond et al., 2008; Adibi, 2019; Warren et al., 2021).

In conclusion, alterations in sensory behaviors, particularly in tactile sensory behaviors, are arguably one of the most significant yet ambiguous phenotypes of ASD. In a condition that truly exists as a spectrum, and is in large part defined by its symptomatic variability, a surprising 90% of individuals with ASD present with sensory deficits (Robertson and Baron-Cohen, 2017). These deficits negatively impact individual’s quality of life and can worsen existing ASD pathology (Orefice et al., 2016). Despite the importance of sensory abnormalities to ASD, there is a surprising lack of sensory behavioral tasks in murine models, limiting their elucidation and consequently, the potential treatment options available. The SNAP methodology addresses this need and is unparalleled in its ease of use, efficiency, and sensory specificity. Since tactile sensory abnormalities are not specific to ASD, SNAP can also be used to assess tactile sensory preferences in murine models of other conditions with tactile sensory alterations such as attention deficit/hyperactivity disorder (ADHD), cerebral palsy, and obsessive-compulsive disorder, among others (Hazen et al., 2008; Cascio, 2010). Altogether, we believe that our novel task will allow researchers to easily assess tactile sensory preferences in addition to locomotion and stereotypy in mice. In addition, the sensory stimuli and the task itself can be readily modified to better distinguish any confounding effects of novelty or anxiety versus sensory abnormalities on the sensory preference.

## Synthesis

Reviewing Editor: David Franklin, Technische Universitat Munchen Decisions are customarily a result of the Reviewing Editor and the peer reviewers coming together and discussing their recommendations until a consensus is reached. When revisions are invited, a fact-based synthesis statement explaining their decision and outlining what is needed to prepare a revision will be listed below. The following reviewer(s) agreed to reveal their identity: NONE.

Your manuscript has been re-reviewed by the two expert reviewers. While both reviewers are generally positive about the manuscript, they have raised several key concerns that will need to be addressed. Overall, they highlighted the significant improvements from the original manuscript and the inclusion of two new strains of mice. However, we have concerns about the interpretation of the findings, mislabelling of the smooth and rough bars in the figure, and highlight concerns about the over-estimation of the value in the test for detecting ASD-like phenotypes in the discussion. In addition to the points highlighted in the individual reviews below, we would like to see much more details about alternative hypotheses for the explanation of your results (more critical and nuanced explanations) and discuss the limitations of the task that you have developed. I encourage you to revise your manuscript while taking into account all of the concerns of the reviewers, and preparing a point-by-point response.

Reviewer 1

The revised version of this manuscript has been greatly improved, and now includes CD-outbred stock and Cntnap2 knockout mice, new approaches for statistical analysis, and an intriguing interpretation of the findings as reflecting sensation-seeking behavior. One remaining concern is that some claims made about this new assay have not yet been substantiated with supporting evidence. For example, the authors note that “SNAP utilizes natural mouse behaviors to create an accurate, reliable, efficient, and precise assessment of somatosensation...”. However, the findings, so far, indicate only that the test can measure a single aspect of somatosensation: increased preference for complex tactile stimulation. It still remains to be determined if this increased preference is due to hyposensitivity of tactile function, relevant to sensory phenotypes in ASD, or to some other type of abnormality, such as stimulation-induced repetitive behavior.

Similarly, the Discussion notes “Thus, our results on tactile sensory preferences are consistent with the clinical ASD literature, exemplifying SNAP’s utility.” Hypersensitivity to environmental stimuli is also a common issue in ASD, and as yet, there is no indication that the SNAP can provide an index of this somatosensory phenotype. Overall, the authors have developed a novel task that has a promising potential, but they should moderate such conclusions as “ Our novel task detected significant sensory abnormalities in ASD-like mice when compared to control mice, showing strong face validity to diagnostic tactile sensory alterations that are observed in individuals with ASD.”

The authors should use caution in their description of the strains as control groups. While C57 mice are typically used as controls for BTBR, the appropriate wild-type controls for the Cntnap2 KO group would be littermate +/+ mice, rather than a set of C57 mice generated from a separate breeding colony, and tested months before the mutant mice. The Discussion indicates: “CNTNAP2 mice were indistinguishable from controls in measures of stereotypy and anxiety.” The wording should be changed from “controls” to “C57 mice,” to clarify the comparison is to an inbred strain, and not a true wild-type group. Similarly, it would be better not to use the confusing term “control CD-1 mice”, but just “CD-1 mice.”

Minor comments:

The correct format for referencing a human gene is all letters capitalized and in italics; for a mouse gene, only the first letter is capitalized, with all letters in italics.

“Significant Statement” should be “Significance Statement”

As noted in the first critique, the formal nomenclature of “BTBR” should be given, preferably in the Subjects section. Just to be clear, BTBR is a short version, similar to the way that C57 is a short version of C57BL/6J. The authors could find the full name of BTBR on the Jackson Laboratory website, or by looking at the titles of Amodeo et al. 2012; Scattoni et al., 2008; or Probbe et al., 2011, in the reference section of the manuscript.

Methods, under Subjects, should include information on the background strain and genetic alteration of the Cntnap2 group.

21 C57BL/6J mice were used. It is not clear how this number was evenly divided between males and females.

Reviewer 2

I appreciated the effort to extend the behavioural analyses to more ASD-model and “normotypical” mouse strains. Indeed CNTNAP2 mice, as BTBR, do show a nosepoking preference for those holes with sandpaper, not for those without.

My enthusiasm towards this task, however, continues to be limited. I agree with authors that this is fast (5 min) and easy to implement, but, this simplicity also have some limitations, and what actually the task is able to identify remains unclear. Control (outbred CD1 and C57 mice) do not respond differently to hole with or without sandpaper, BTBR and CNTNAP2 mice do prefer nosepoking in holes with sandpaper.

Is preference for rough condition enough for stating that there is a sensory deficit?

Is it a sign of sensation seeking behaviour?

Moreover, these holes are only in the central part of the platform and not uniformly distributed so it is difficult to disentangle preference (or aversion) for this area with number of nose poking visits. Indeed, how can authors exclude that BTBR mice do make more visits just because they stay longer in the area where holes are? The argument that this does not entirely apply to CNTNAP2 mice does not necessarily means that this is also true for BTBR. By contrast, they could end up to make more visits just because the holes are only in the central area (or viceversa). And the opposite could be true for C57 or Cd-1 mice.

The authors state that the task they developed is fast and effective; as first screening it could probably be interesting, but more awareness on limitations and uncertainties will render the reading of their article more useful for people interested in wider behavioural phenotyping of ASD mouse models.

I would list main effect first (texture and strain), then interactions (texture x strain).

Sidak post hoc analyses is mentioned in the results, Tukey HSD in the statistical section.

Importantly, Figure 2 does show preference for smooth condition in both BTBR and CNTNAP2 mice, the opposite of what described in the text.

## Author Response

Response to reviewers

We appreciate the reviewers’ and editor’ time to re-review our manuscript. Based on the reviewer’s questions and suggestions, we significantly rewrote the results and discussion of the manuscript to not oversell the newly developed task. We also emphasized the weaknesses and highlight improvements that could be made or conditions that could be tested. I hope this will satisfy the reviewers. The specific responses are outlined point-by-point below and changes are highlighted blue in the revised manuscript. A clean version of the manuscript is also provided.

General comment from the editor:

Your manuscript has been re-reviewed by the two expert reviewers. While both reviewers are generally positive about the manuscript, they have raised several key concerns that will need to be addressed. Overall, they highlighted the significant improvements from the original manuscript and the inclusion of two new strains of mice. However, we have concerns about the interpretation of the findings, mislabelling of the smooth and rough bars in the figure, and highlight concerns about the over-estimation of the value in the test for detecting ASD-like phenotypes in the discussion. In addition to the points highlighted in the individual reviews below, we would like to see much more details about alternative hypotheses for the explanation of your results (more critical and nuanced explanations) and discuss the limitations of the task that you have developed. I encourage you to revise your manuscript while taking into account all of the concerns of the reviewers, and preparing a point-by-point response.

Response: Thank you for the opportunity to resubmit. We fixed figure 3, the results section, and significantly amended the discussion as suggested. In particular, we provided additional data interpretations and ways to improve the described behavioral task. We also considerably softened our claims and changed the title.

Reviewer 1

Comment 1. The revised version of this manuscript has been greatly improved, and now includes CD-outbred stock and Cntnap2 knockout mice, new approaches for statistical analysis, and an intriguing interpretation of the findings as reflecting sensation-seeking behavior. One remaining concern is that some claims made about this new assay have not yet been substantiated with supporting evidence. For example, the authors note that “SNAP utilizes natural mouse behaviors to create an accurate, reliable, efficient, and precise assessment of somatosensation...”. However, the findings, so far, indicate only that the test can measure a single aspect of somatosensation: increased preference for complex tactile stimulation. It still remains to be determined if this increased preference is due to hyposensitivity of tactile function, relevant to sensory phenotypes in ASD, or to some other type of abnormality, such as stimulation-induced repetitive behavior.

Response: I apologize for the overinterpretation of our data. Indeed, the task cannot distinguish the different aspects of somatosensation. We thus changed the text (in abstract, end of introduction, and discussion) to reflect that our task only assessed increased preference for complex tactile stimulation. In addition, as outlined by the reviewer, we do not know the exact somatosensory mechanism (e.g., hyposensitivity of tactile function or increased stimulation-induced repetitive behavior) responsible for this preference. This has been included in the discussion.

Comment 2: Similarly, the Discussion notes “Thus, our results on tactile sensory preferences are consistent with the clinical ASD literature, exemplifying SNAP’s utility.” Hypersensitivity to environmental stimuli is also a common issue in ASD, and as yet, there is no indication that the SNAP can provide an index of this somatosensory phenotype. Overall, the authors have developed a novel task that has a promising potential, but they should moderate such conclusions as “ Our novel task detected significant sensory abnormalities in ASD-like mice when compared to control mice, showing strong face validity to diagnostic tactile sensory alterations that are observed in individuals with ASD.”

Response: We agree with the reviewer that the tactile sensory abnormalities in humans with ASD are complex and cannot be fully reflected by our task. I changed the notes at the end of the introduction and in the discussion that were related to the applicability of the novel task. In the introduction, we removed the reference to humans with ASD.

Comment 3: The authors should use caution in their description of the strains as control groups. While C57 mice are typically used as controls for BTBR, the appropriate wild-type controls for the Cntnap2 KO group would be littermate +/+ mice, rather than a set of C57 mice generated from a separate breeding colony, and tested months before the mutant mice. The Discussion indicates: “CNTNAP2 mice were indistinguishable from controls in measures of stereotypy and anxiety.” The wording should be changed from “controls” to “C57 mice,” to clarify the comparison is to an inbred strain, and not a true wild-type group. Similarly, it would be better not to use the confusing term “control CD-1 mice”, but just “CD-1 mice.”

Response: This is a good point. We removed the word “control” and replaced with neurotypical. We also removed “control” in front of CD-1 mice.

Minor comments:

Comment 1: The correct format for referencing a human gene is all letters capitalized and in italics; for a mouse gene, only the first letter is capitalized, with all letters in italics.

Response: Thank you. We corrected when applicable.

Comment 2: “Significant Statement” should be “Significance Statement”

Response: This has been correct.

Comment 3: As noted in the first critique, the formal nomenclature of “BTBR” should be given, preferably in the Subjects section. Just to be clear, BTBR is a short version, similar to the way that C57 is a short version of C57BL/6J. The authors could find the full name of BTBR on the Jackson Laboratory website, or by looking at the titles of Amodeo et al. 2012; Scattoni et al., 2008; or Probbe et al., 2011, in the reference section of the manuscript. -

Response: The proper names have been listed in the Subjects section as suggested.

Comment 4: Methods, under Subjects, should include information on the background strain and genetic alteration of the Cntnap2 group.

Response: The full name, which includes the background strain, was added.

Comment 5: 21 C57BL/6J mice were used. It is not clear how this number was evenly divided between males and females.

Response: Thank you for this comment. We broke down male and female numbers by strain in Subjects section.

Reviewer 2

General comment: I appreciated the effort to extend the behavioural analyses to more ASD-model and “normotypical” mouse strains. Indeed CNTNAP2 mice, as BTBR, do show a nosepoking preference for those holes with sandpaper, not for those without.

My enthusiasm towards this task, however, continues to be limited. I agree with authors that this is fast (5 min) and easy to implement, but, this simplicity also have some limitations, and what actually the task is able to identify remains unclear. Control (outbred CD1 and C57 mice) do not respond differently to hole with or without sandpaper, BTBR and CNTNAP2 mice do prefer nosepoking in holes with sandpaper.

Response: We apologize for over-interpretating our data and overselling our task. We agree that our simple task may have additional interpretations and could be modified to better assess sensory preferences or abnormalities. Our goal was to describe a simple, baseline task that can be used in combination with others to identify behavioral alterations in ASD mouse models. To acknowledge both reviewers’ comments, we have provided alternate interpretations of the data as well as suggesting ways to improve the task. This is included in the abstract and the discussion.

Comment 1: Is preference for rough condition enough for stating that there is a sensory deficit?

Comment 2: Is it a sign of sensation seeking behaviour?

Response: We had indeed provided only one single interpretation of our data mentioning that the observed behavior suggested a sensory preference and a sensation seeking behavior based on the increased preference for complex tactile stimulation. But, as mentioned above by both reviewers, there are certainly other interpretations of the data. One alternate interpretation is that the rough condition may represent novelty and thus animals will prefer the novel rough condition that they have not been exposed to. Anxiety could also be triggered or aggravated by the openness of the arena and affect sensory preferences.

We thus discussed these shortcomings and provide options to mitigate novelty and anxiety (significant rewrite of the discussion). We softened our conclusions in both the discussion and the abstract. We also change the title to better fit the new data interpretation.

Comment 3: Moreover, these holes are only in the central part of the platform and not uniformly distributed so it is difficult to disentangle preference (or aversion) for this area with number of nose poking visits. Indeed, how can authors exclude that BTBR mice do make more visits just because they stay longer in the area where holes are? The argument that this does not entirely apply to CNTNAP2 mice does not necessarily means that this is also true for BTBR. By contrast, they could end up to make more visits just because the holes are only in the central area (or viceversa). And the opposite could be true for C57 or Cd-1 mice.

Response: We agree with the reviewer. The argument based on CNTNAP2 data may not apply to BTBR mice. It is possible that BTBR mice stay preferentially in the middle and then have more opportunities for visiting the holes. We thus modified our discussion highlighting this limitation of our task and propose to have the holes uniformly distributed throughout the platform as suggested by the reviewer.

Comment 4: The authors state that the task they developed is fast and effective; as first screening it could probably be interesting, but more awareness on limitations and uncertainties will render the reading of their article more useful for people interested in wider behavioural phenotyping of ASD mouse models.

Response: We totally agree. We added discussion on the limitations and uncertainties about the task as pointed out by both reviewers.

Comment 5: I would list main effect first (texture and strain), then interactions (texture x strain).

Sidak post hoc analyses is mentioned in the results, Tukey HSD in the statistical section.

Response: We rewrote the statistical analysis in the results section in the order suggested. We are sorry for the confusion regarding the post-hoc analyses. We erroneously wrote Sidak instead of Tukey HSD for Figure 3 data that used one way ANOVA. This has been corrected in the Results section. As mentioned in the Methods section, we used Sidak post-hocfor repeated measures ANOVA and Tukey HSD for one way ANOVA.

Comment 6: Importantly, Figure 2 does show preference for smooth condition in both BTBR and CNTNAP2 mice, the opposite of what described in the text.

Response: Thank you very much for catching this error. It is now fixed.
